# The study of plasmodesmal biology using proximity labeling technologies

**DOI:** 10.1093/jxb/eraf239

**Published:** 2025-05-30

**Authors:** Zhongpeng Li, Kyaw Aung

**Affiliations:** Department of Genetics, Development, and Cell Biology, Iowa State University, Ames, IA 50011, USA; Department of Genetics, Development, and Cell Biology, Iowa State University, Ames, IA 50011, USA; Donald Danforth Plant Science Center, USA

**Keywords:** PD proteome, plasmodesmata (PD), proximity labeling (PL), TurboID (TbID)

## Abstract

Plasmodesmata (PD) are essential cellular structures that facilitate intercellular communication in plants, enabling the transport of nutrients and signaling molecules. Over the past decades, significant strides have been made in unraveling the formation, function, and regulation of PD. Identification and functional characterization of PD-associated proteins have greatly advanced our understanding of PD. This review discusses past efforts in uncovering PD proteomes and highlights recent breakthroughs in applying proximity labeling (PL) assays to map plant protein interactomes. Special attention is given to using PL assays in studying PD biology, emphasizing their potential to drive future advancements and deepen our understanding of PD function and regulation. By integrating PL technologies with established methodologies, researchers can gain comprehensive insights into the dynamic composition and roles of PD.

## Teaser

Implementation of proximity labeling technologies will advance our understanding of plasmodesmata.

## Introduction

Plasmodesmata (PD) are nanoscopic pores that traverse plant cell walls, creating continuity of the cytoplasm, plasma membrane (PM), and endoplasmic reticulum (ER) of neighboring cells. These structures establish the symplast in plants, facilitating cell-to-cell communication and enabling the exchange of nutrients and information between adjacent cells and distant tissues (Bayer and Benitez-Alfonso, 2024). By mediating the transport of ions, metabolites, proteins, and RNA molecules, PD play critical roles in plant growth, development, and stress responses (Miras *et al*., 2022; Tee and Faulkner, 2024). The essential nature of PD is underscored by the severe consequences of their structural defects or functional impairments, which can lead to embryo lethality or profound molecular and pleiotropic phenotypes (Brunkard and Zambryski, 2017).

PD are highly dynamic and complex structures with diverse molecular components (Z.P. Li *et al*., 2021). A wide array of proteins localize to distinct PD subdomains, including the PM within PD (PD-PM), the ER within PD (PD-ER), and the surrounding cell wall (CW), constituting the PD proteome. Additionally, proteins that stably or transiently occupy the cytoplasmic sleeve, the space between the PD-PM and PD-ER, are also considered part of the PD proteome. Proteomic analyses have led to the identification and functional characterization of multiple key PD-associated proteins, such as β-1,3-GLUCANASES (Levy *et al*., 2007), the glycophosphatidylinositol (GPI)-anchored receptor LYSIN MOTIF-CONTAINING GPI-ANCHORED PROTEIN 2 (LYM2) (Faulkner *et al*., 2013), and MULTIPLE C2 DOMAINS TRANSMEMBRANE PROTEINS (MCTPs) (Brault *et al*., 2019). Functional characterization of β-1,3-GLUCANASES has shown that these enzymes degrade callose deposited at PD, thereby modulating PD permeability and function (Levy *et al*., 2007). MCTPs are associated with the PD-ER and interact with the PD-PM to regulate PD function in a callose-independent manner (Pérez-Sancho *et al*., 2025). Furthermore, MCTPs play essential roles in PD formation during cell division (Z.P. Li *et al*., 2024). The study of LYM2 revealed a distinct immune signaling pathway at PD involving calcium-dependent protein kinases, NADPH oxidase, PLASMODESMATA-LOCATED PROTEINS (PDLPs), NON-RACE SPECIFIC DISEASE RESISTANCE/HIN1 HAIRPIN-INDUCED-LIKE 3 (NHL3), and CALLOSE SYNTHASE1 (CALS1). Upon sensing chitin, the LYM2-mediated signaling cascade promotes callose accumulation at PD, thereby regulating intercellular communication during microbial infection (Faulkner *et al*., 2013; Cheval *et al*., 2020; Tee *et al*., 2023). Collectively, these findings demonstrate how the identification and functional analysis of PD-associated proteins provide critical insights into PD regulation and function.

## Identification of the plasmodesmal proteome

PD are structurally complex and challenging to isolate, making the identification of their molecular components a technically demanding task. Over the past decades, significant progress has been made in identifying PD components and elucidating their roles. Several strategies have been employed to map the PD proteome, ranging from early biochemical fractionation techniques to advanced quantitative proteomics and computational predictions (Box 1). Early successes in mapping the PD proteome began with purifying cell walls and isolating PD-enriched fractions coupled with MS using Arabidopsis (Thomas *et al*., 2008; Fernandez-Calvino *et al*., 2011). Subsequent studies refined the PD purification technique and incorporated comparative proteomic quantification, improving the identification of PD proteomes in Arabidopsis (Kraner *et al*., 2017; Brault *et al*., 2019), *Nicotiana benthamiana* (Park *et al*., 2017), and *Populus trichocarpa* (Leijon *et al*., 2018). Integrative approaches that combine experimental proteomics with computational analyses have significantly enhanced the reliability and comprehensiveness of PD proteomes (Gombos *et al*., 2023; Johnston *et al*., 2023). Leveraging four published experimental PD proteomes from Arabidopsis, *N. benthamiana*, and *Populus*, along with the PD proteins with experimentally verified localization in Arabidopsis, Kirk *et al*. (2022) developed an *in silico* PD proteome prediction pipeline that uses protein subfamily classifications and structural features to predict PD-associated proteins across diverse plant species. Immunoprecipitation (IP) followed by MS (IP-MS) analysis was adopted to identify putative partners of PDLP1 (Caillaud *et al*., 2014), PDLP7 (Chen *et al*., 2024), RETICULONs (Kriechbaumer *et al*., 2015), CYSTEINE-RICH RECEPTOR KINASE 2 (CRK2; Hunter *et al*., 2019), and FRUIT WEIGHT 2.2 (FW2.2; Beauchet *et al*., 2024). Using a split-ubiquitin yeast two-hybrid assay, NON-RACE SPECIFIC DISEASE RESISTANCE/HIN1 HAIRPIN-INDUCED-LIKE PROTEIN 3 (NHL3) was identified as an interacting protein of PDLP5, regulating callose accumulation and PD function (Tee *et al*., 2023). The identification and functional characterization of PD-associated proteins have greatly expanded our understanding of the function and regulation of PD.

Box 1. **Key developments in identifying the PD proteome**

Purifying cell walls and isolating PD-enriched fractions coupled with MS

Thomas *et al*. (2008) identify cell wall-associated membrane proteins and characterized PD-localized proteins. Fernandez-Calvino *et al*. (2011) report the first PD proteome by purifying PD from the walls of Arabidopsis suspension cells. Kraner *et al*. (2017) report a comparative proteomic approach using Arabidopsis leaves to identify components of complex PD.

Park *et al*. (2017) identify proteins in PD-enriched cell wall fractions from mock and turnip mosaic virus-infected *Nicotiana benthamiana*. Leijon *et al*. (2018) identify proteins in PD-enriched cell wall fractions from cell suspension cultures of *Populus trichocarpa*. Brault *et al*. (2019) refine the PD proteome in Arabidopsis suspension culture cells using a refined PD purification technique and label-free comparative quantification.

Integrative approaches

Johnston *et al*. (2023) generate two novel PD proteomes from mature Arabidopsis leaves and *Physcomitrium patens* and use phyloproteomic comparison to define a more detailed atlas of PD structure and function. Gombos *et al*. (2023) define a high-confidence PD proteome using an iterative scoring system in *Physcomitrium patens*.

*In silico* prediction

Kirk *et al*. (2022) report *in silico* prediction of PD proteomes based on a meta-analysis that leverages the conserved structural features of verified PD proteins.

Identification of PD-localized protein interactors

Caillaud *et al*. (2014) identify PDLP1-interacting proteins in downy mildew-infected and uninfected Arabidopsis tissues using an immunoprecipitation approach. Kriechbaumer *et al*. (2015) investigate the binding partners of two PD-resident reticulon proteins, RTNLB3 and RTNLB6, by co-immunoprecipitation followed by MS. Hunter *et al*. (2019) identify CRK2-interacting proteins from Arabidopsis seedlings by immunoaffinity purification followed by MS. Tee *et al*. (2023) conduct a split-ubiquitin screen of an Arabidopsis cDNA library to identify PDLP5-interacting proteins. Z. Li *et al*. (2024) apply a proximity labeling assay to identify Arabidopsis PDLP5, PDLP6, and MCTP3 protein partners. Chen *et al*. (2024) identify Arabidopsis PDLP7-interacting proteins through an immunoprecipitation assay using purified PD fractions and a PDLP7-specific antibody.

## Application of proximity labeling to study plasmodesmal proteins

Proximity labeling (PL) assay is a biochemical technique that uses enzyme-based labeling to tag neighboring endogenous molecules within nanometer proximity of a bait protein (Niinae *et al*., 2021; Qin *et al*., 2021). PL enzymes, such as biotin ligases [e.g. TurboID (TbID)], peroxidases (e.g. APEX2), and Pup ligase, fused to a protein of interest can covalently modify nearby proteins. Labeled proteins are subsequently purified and identified using MS. Biotin ligases, such as BioID, TbID, miniTurbo, and split-TbID, along with the pupylation-based interaction tagging (PUP-IT) system, have become valuable tools for studying protein interactomes and local proteomes in plants (Yang *et al*., 2020; Mair and Bergmann, 2022; Xu *et al*., 2023).

### Application of PL to study plant biology

The past 2 years have witnessed a surge of reports on applying PL in plants. These methods have been widely adopted to investigate diverse biological processes such as pathogen infection (Conlan *et al*., 2018; Khan *et al*., 2018; Zhang *et al*., 2019; Chicowski *et al*., 2023 , Preprint; Gryffroy *et al*., 2023; Zhang *et al*., 2024), stress responses (Van Leene *et al*., 2022; Zhou *et al*., 2023; Bao *et al*., 2024 ), hormone signaling (Kim *et al*., 2023 ; Yang *et al*., 2023; Chien *et al*., 2024), plant development (Mair *et al*., 2019 ; Barbosa *et al*., 2023; Feng *et al*., 2023; Liu *et al*, 2024; Wallner *et al*., 2024), and subcellular proteomes (Tang *et al*., 2020, 2024; Jia *et al*., 2023 ). PL has been adapted to a variety of plant species, including Arabidopsis, *N. benthamiana*, maize (L. Shi *et al*., 2023), soybean (Chicowski *et al*., 2023, Preprint; Margets *et al*., 2024), rice (Lin *et al*., 2017), potato (W. Shi *et al*., 2023), tomato (Gryffroy *et al*., 2023; De Ryck *et al*., 2025), petunia (Aravena-Calvo *et al*., 2024), liverwort (Melkonian *et al*., 2022), and green algae (Kreis *et al*., 2023 ; Lau *et al*., 2023), highlighting its versatility across model systems, crops, and non-seed plants. These studies underscore the transformative potential of PL technologies in advancing plant science, offering an efficient way to map proteins localized to different subcellular compartments and involved in diverse biological processes.

### Application of PL to study PD biology

PD are intricately embedded in the extracellular matrix without distinct boundaries. Thus, purifying PD from cell wall fractions without contaminants from other membranes is notoriously difficult. PD proteins could reside on PD-PM, PD-ER, or in the CW. In addition, many proteins in the cytoplasmic sleeve probably transiently or weakly attach to PD. These characteristics make it difficult to comprehensively map the PD proteome using traditional techniques.

Advancements in PL technologies have provided an unprecedented opportunity to identify novel PD proteins. Recently, TbID was used to identify protein partners of PD-associated proteins, including PDLP5, PDLP6, and MCTP3 (Z. Li *et al*., 2024). PDLP5 and PDLP6 are localized to the PD-PM (Thomas *et al*., 2008; Lee *et al*., 2011), whereas MCTP3 is targeted to the PD-ER (Brault *et al*., 2019). This study expressed PDLP5–TbID, PDLP6–TbID, and TbID–MCTP3 in Arabidopsis, with the TbID domain facing the cytoplasmic sleeve (Z. Li *et al*., 2024). PDLP5–TbID and PDLP6–TbID significantly enriched PD- and PM-associated proteins compared with MCTP3, while TbID–MCTP3 enriched numerous ER- and PD-ER-associated proteins (Z. Li *et al*., 2024). The identification of PD-ER proteins by TbID–MCTP3 reinforces the power of the PL assay in identifying the PD proteome at the precise subcompartment. Additionally, the results also imply that PDLP5–TbID and PDLP6–TbID identify bona fide PD-PM proteins (Z. Li *et al*., 2024).

Although PDLP5–TbID and PDLP6–TbID yielded largely overlapping proteins, they also identified distinct subsets of proteins when directly compared with each other. For instance, SUCROSE SYNTHASE 6 (SUS6) was significantly enriched by PDLP6–TbID compared with PDLP5–TbID, while PDLP5–TbID significantly enriched several members of the PLASMA MEMBRANE INTRINSIC PROTEINS (PIPs) (Z. Li *et al*., 2024). Together, these findings demonstrate the capability of PL assays to resolve protein complexes at PD with nanometer-scale resolution. Notably, despite using a constitutive promoter, *pUBQ10*, PDLP6–TbID significantly enriched a cell-type-specific functional partner, SUS6, compared with PDLP5–TbID (Z. Li *et al*., 2024). Together, these findings highlight how different comparative analyses or choices of controls can yield different candidate lists, reinforcing the critical importance of proper controls in PL assays.

While PL is a powerful tool for mapping PD proteomes, it also comes with certain challenges. Table 1 summarizes the principles, advantages, and challenges of the various strategies used to identify PD proteomes.

**Table 1.** Comparison of methods for identifying PD proteomes

Method	Principles	Advantages	Challenges
*In silico* prediction	Computational prediction based on sequence motifs, structural features, or homology to know PD proteins.	High throughput without high experimental costs. Can identify novel candidates. Can easily apply to different plant species.	Requires experimental validation. False positive/negative due to algorithm limitations.
PD purification and MS	Isolation of PD fractions biochemically, followed by MS analysis.	Enriches PD-associated proteins. Provides direct experimental evidence. Not required to generate transgenic plants. Enable the direct comparison between wild-type and mutant plants.	Challenging isolation process. Contamination from non-PD components. Involves extensive laboratory experimentation.
Immunoprecipitation and MS	Precipitation of PD-associated protein complexes using an antibody, followed by MS analysis.	Identifies protein complexes and physical interactors. Identifies PD proteomes in response to stresses.	Requires generating genetic materials, including transgenic plants. Requires confirming the PD localization and function of fusion proteins if an epitope is used. Misses weak and/or transient interactions. Involves extensive laboratory experimentation.
Proximity labeling assay and MS	Fusion of a biotin ligase (e.g. TurboID) to a PD protein enables biotinylation of nearby proteins, followed by the enrichment of biotinylated proteins and MS analysis.	Identifies protein complexes and physical interactors at a much higher specificity. Identifies PD proteomes in response to stresses. Identifies PD proteomes at higher spatial and temporal resolutions. Identifies cell type-specific PD proteomes. Identifies weak and/or transient interactions.	Requires generating genetic materials, including transgenic plants. Requires transgenic plants with good biotinylation efficiency. Requires confirming the PD localization and function of biotin ligase fusion proteins. Identifies false positives due to promiscuous biotinylation of nearby, non-specific proteins. Involves extensive laboratory experimentation.

## Optimization of proximity labeling experiment design to map plasmodesmal proteomes

To improve the accuracy and reliability of PL assays for studying PD-associated proteins—and to further expand its applications in plant research—careful consideration of key experimental designs and validation strategies is essential.

### Validating PD localization and function of TbID fusion proteins

The fusion of PL enzymes with PD proteins can potentially alter their function, localization, or interactome. Therefore, confirming the PD localization and functionality of TbID fusion proteins is essential. *Agrobacterium*-mediated transient expression in *N. benthamiana*, combined with live-cell imaging and immunoblot analysis, provides a reliable and efficient approach to verifying the PD localization and enzymatic activity of TbID fusion proteins. If transgenic plants are used for PD proteome mapping, it is crucial to confirm the expression and activity of the fusion protein in these plants to ensure the selection of appropriate lines for further studies. Additionally, complementing a knockout mutant with the corresponding TbID fusion protein offers a more stringent strategy to further validate its function, ensuring that the fusion of TbID does not compromise the native activity of the PD protein.

### Choosing the optimal promoters for fusion protein expression

Selecting the appropriate promoter for fusion protein expression is crucial. It should align with the primary objective of the experiment, the expression level of the protein of interest, and its cell or tissue specificity (Xu *et al*., 2023). While native promoters are often preferred for preserving physiological relevance, they may pose challenges in identifying interactomes if the protein of interest is expressed at low levels or restricted to specific cell types. The *UBQ10* promoter has been successfully employed to identify protein interactomes of several PD proteins, including PDLP5, PDLP6, and MCTP3 (Z. Li *et al*., 2024). Notably, this approach led to the identification of SUS6, a cell-type-specific partner of PDLP6 (Z. Li *et al*., 2024). To enhance the likelihood of detecting protein partners, strategies such as enriching specific cell types to boost TbID fusion protein levels or using a promoter with higher expression in the target cell type can be effective. The recent advancements in single-cell RNA sequencing provide a valuable resource for selecting cell-type-specific promoters with higher expression levels (Kim *et al*., 2021; Seyfferth *et al*., 2021; Shahan *et al*., 2022; Cao *et al*., 2024; He *et al*., 2024), thereby improving the detection of genuine interaction partners within defined cell types.

### Including appropriate controls

Due to background biotinylation and the labeling of proximal proteins beyond direct protein partners, including appropriate controls is essential. The choice of controls may vary depending on the experimental needs, with different plant genotypes or treatments serving as useful comparisons. For example, Arabidopsis wild-type Col-0 can be used to establish baseline biotinylation levels. In our view, Col-0 serves as an excellent control when the objective is to identify a broad range of candidate proteins rather than to resolve protein complexes at high resolution. Incorporating TbID-tagged free soluble proteins, such as green fluorescent protein (GFP), as controls allows for comparing biotinylated candidates between proteins localized to the nucleus and cytosol versus those localized to specific cellular compartments (e.g. PD). To specifically identify protein complexes localized to the PD-PM (e.g. PDLP5), proteins located in other subcellular regions, such as the bulk PM (e.g. RCI2A) (Zhou *et al*., 2020) or PD-ER (e.g. MCTP3) (Brault *et al*., 2019), can serve as controls. If the aim is to map protein complexes within subdomains at the same membrane compartment (e.g. PD-PM), controls such as PD-PM-localized yellow fluorescent protein (YFP) or other PD-PM-targeted proteins (e.g. PDLPs, REMORINs, and SRF3) (Thomas *et al*., 2008; Huang *et al*., 2019; Platre *et al*., 2022) are ideal choices. In cases where the goal is to identify protein partners enriched by stress, transgenic lines treated with or without the stress will help identify proteins specifically enriched by the stress condition. When selecting a TbID fusion as a control, choosing one with expression and enzymatic activity levels like those of the protein of interest is preferable. Selecting appropriate controls is essential for the success of experiments with varying objectives.

## Selection and functional characterization of novel plasmodesmal proteins

### Candidate selection

PL identifies proteins in the proximity of a bait protein, capturing direct interactors, indirect interactors, and nearby proteins, potentially identifying several hundred candidates. Different criteria and parameters can be applied to select or prioritize the candidate protein. If PD-PM proteins were used as baits, the top candidates to validate would be those probably targeted to or associated with the PD-PM. If TbID is tagged to the cytosolic tail of PD-PM proteins, the candidate proteins localized to PD-PM or the cytosol would be a proper candidate rather than the proteins targeted to the apoplast or other cellular compartments (e.g. the mitochondrial matrix). Another major criterion for top candidates would be those with potential functions in the same cellular processes as the bait proteins. For example, selecting SUS6 as a functional partner of PDLP6 makes logical sense as they are both specifically expressed in the vasculature and have a potential role in synthesizing callose (Z. Li *et al*., 2024).

If the structure of the bait and candidate proteins can be predicted by artificial intelligence (AI)-based protein structure prediction tools, such as AlphaFold, with high confidence, the utilization of AlphaFold-Multimer (Evans *et al*., 2022, Preprint) and AlphaFold3 (Abramson *et al*., 2024) offers significant advantages in screening for the candidate proteins that physically interact with the bait proteins (Tang *et al*., 2024). These tools can predict complex structures with high accuracy, providing insights into potential binding interfaces. The tools enable further screening of many protein pairs in a short period, making them a great complement to experimental validation methods and generating working hypotheses.

### Determining the physical interactions

The physical interaction between the candidates and the bait proteins will help narrow down the identification of the proteins forming protein complexes with the bait at PD. Yeast two-hybrid (Y2H; Chen *et al*., 2024), split-ubiquitin Y2H (su-Y2H; Tee *et al*., 2023), fluorescence resonance energy transfer (FRET; Wang *et al*., 2020), and bimolecular complementation (BiFC; Wang *et al*., 2020) approaches can be used to determine the direct physical interactions between PD proteins. Co-immunoprecipitation (CoIP; Z. Li *et al*., 2024), on the other hand, enables the determination of the proteins in the same complex. As PL assays can identify candidate proteins that are only transiently associated with the bait proteins, candidate proteins without strong direct or indirect association with the bait might also form a complex with the bait proteins.

### Determining PD localization

Determining the association of candidate proteins with PD will help prioritize those with more direct roles in regulating PD function. Transient expression of fluorescent protein-tagged candidate proteins via *Agrobacterium*-mediated expression is a widely used, efficient, and reliable method for validating PD localization (Aung *et al*., 2017; Gombos *et al*., 2023). Delivery of plasmid DNA via microparticle bombardment could be an alternative approach to transiently express the candidate proteins in plants unsuitable for the *Agrobacterium*-mediated expression approach (Aung *et al*., 2017). PD-associated protein markers such as fluorescently tagged PDLP1 (Thomas *et al*., 2008) or aniline blue staining (Zavaliev and Epel, 2015) can be employed to determine PD co-localization. It is noted that proteins that do not stably localize to PD may still contribute to PD function by interacting with bait proteins or other components involved in PD regulation.

### Functional characterization

Different strategies can be employed to investigate the role of candidate proteins in regulating PD, depending on their potential molecular functions. Characterizing knockout mutants and transgenic plants overexpressing the candidate proteins provides valuable insights into their functions. To assess the involvement of candidate proteins in PD regulation, two fundamental approaches are commonly used: examining PD callose accumulation and evaluating PD-dependent molecular movement. Aniline blue staining (Zavaliev and Epel, 2015; Sankoh *et al*., 2024) and immunolabeling using a commercially available antibody against callose (Pendle and Benitez-Alfonso, 2015) are standard techniques for determining PD callose accumulation. To study PD-dependent molecular movement, methods such as *Agrobacterium*-mediated transient expression (Brunkard and Zambryski, 2019; Z. Li *et al*., 2021), microparticle bombardment (Faulkner *et al*., 2013; Aung *et al*., 2017, 2020), or a Drop-ANd-See method (Cui *et al*., 2015) can be applied.

## Future applications of proximity labeling technologies in studying plasmodesmal biology

**Fig. 1.**
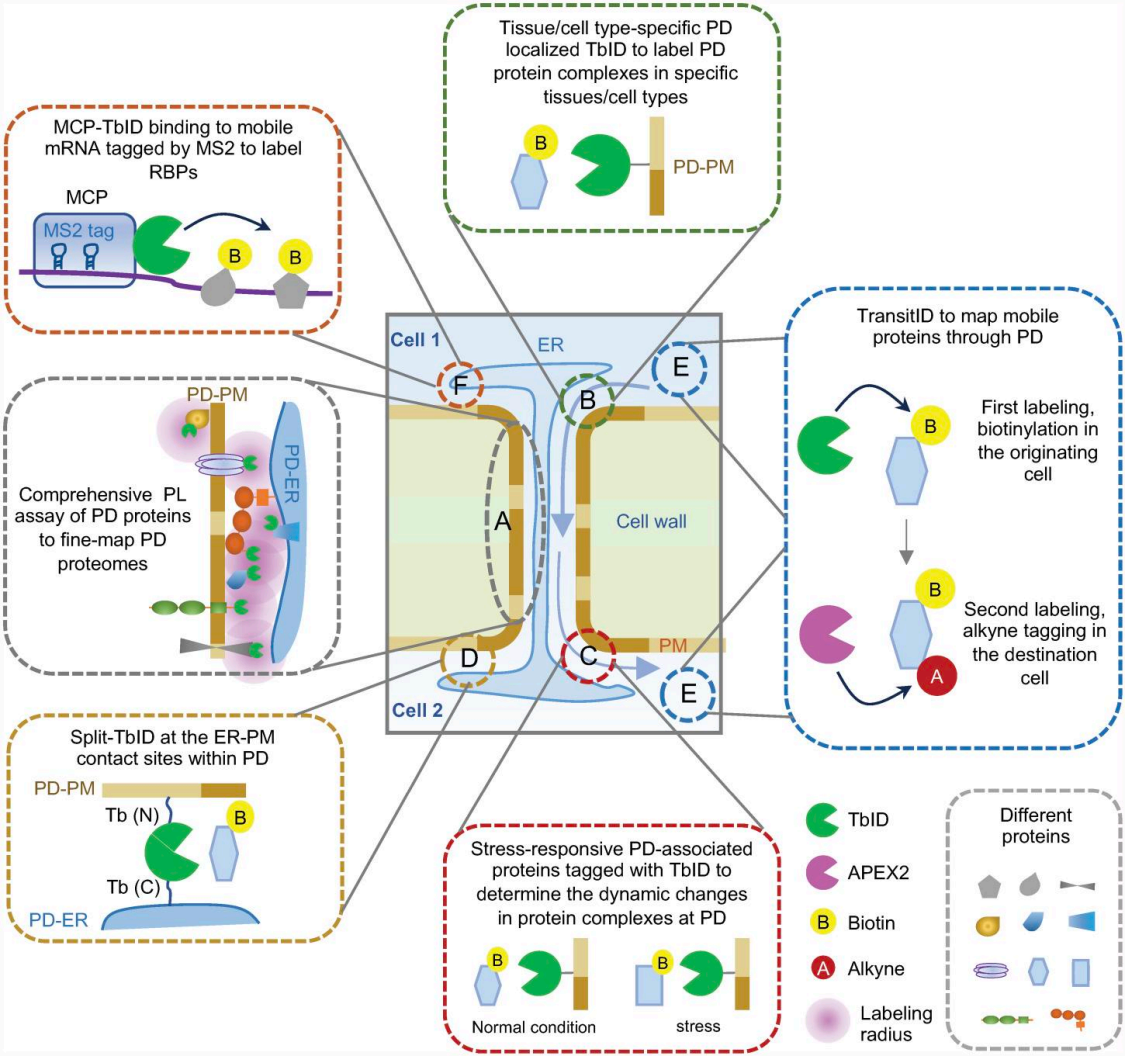
Potential applications of diverse PL technologies in studying PD biology. Various PL technologies have been developed in recent years, offering promising avenues for advancing the study of PD biology. (A) Comprehensive PL assays using known PD-localized proteins as baits to map PD subdomain proteomes. (B) Tissue/cell type-specific PD-localized proteins tagged with TbID to identify PD protein complexes in specific tissues/cell types. (C) Stress-responsive PD-associated proteins tagged with TbID to identify PD protein complexes in response to stresses. (D) Split-TbID to identify proteins at PD-PM and PD-ER contact sites. (E) TransitID to map endogenous protein movement between cells through PD. (F) MS2-based TbID assay to identify RBPs of mobile RNAs.

The original PL technique was developed to map protein interactomes and local proteomes in living cells. New PL techniques have been designed to increase the spatiotemporal specificity and versatility, allowing researchers to tailor the approach to specific experimental needs. In recent years, advancements in PL technologies, such as split-TbID (Cho *et al*., 2020), LOV-Turbo (Lee *et al*., 2023), TransitID (Qin *et al*., 2023), LAccID (Lee *et al*., 2024, Preprint), APEX-seq (Fazal *et al*., 2019), RNA-BioID (Mukherjee *et al*., 2019), BLITZ (Xiong *et al*., 2021), and PUP-IT (Liu *et al*., 2018), have significantly expanded the potential application of PL in plant research (Box 2). Novel PL methods, although primarily developed for other organisms, could be adapted in plants to study PD biology (Fig. 1). Below, we discuss how diverse PL technologies can address key questions about PD composition, dynamics, and function.

Box 2. **Recent advances in proximity labeling technologies**

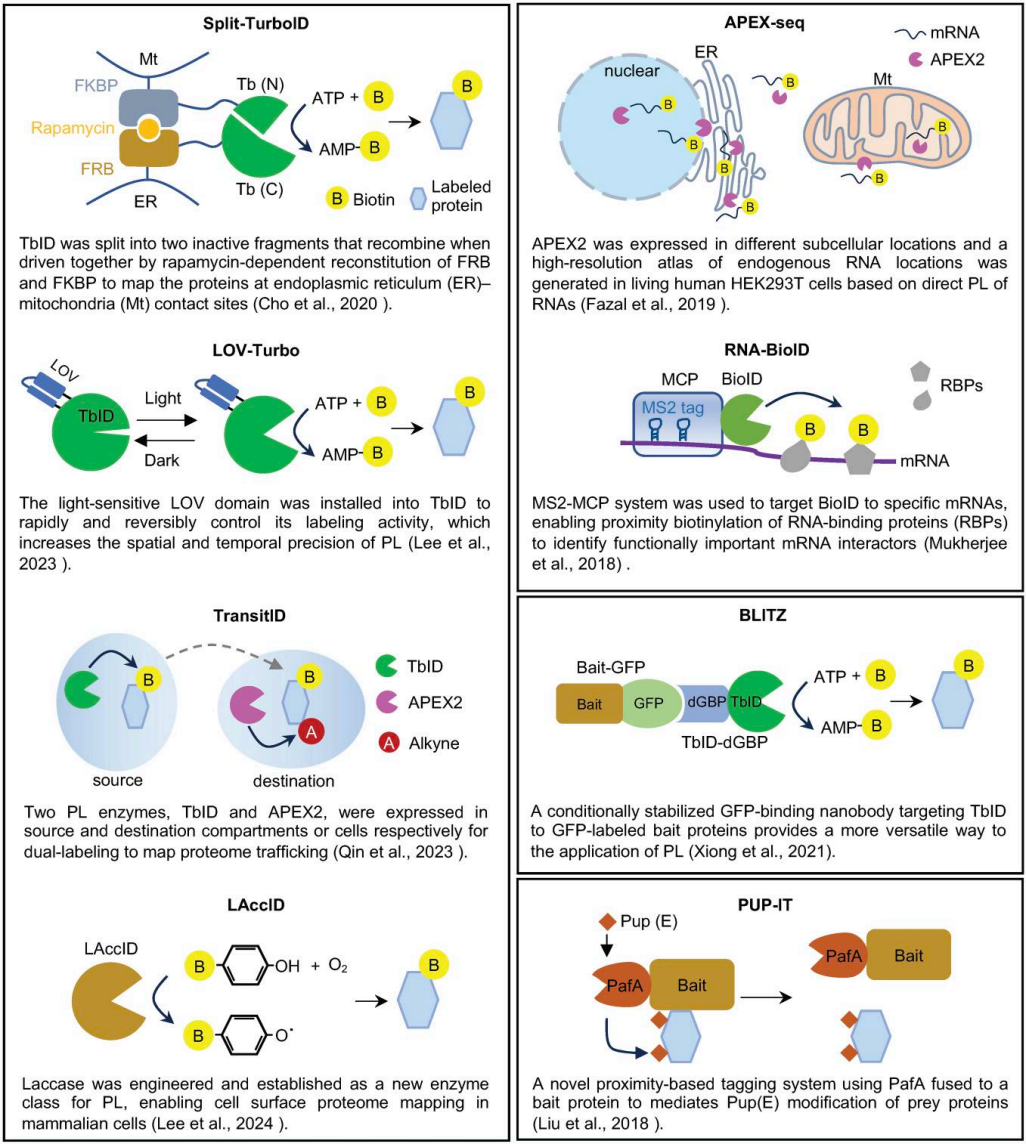

### Mapping protein complexes within PD subdomains

Mapping the protein partners of PD-localized proteins that target specific subdomains within PD will uncover protein complexes at nanometer-scale resolution (Fig. 1A). For instance, PDLPs (Thomas *et al*., 2008), NHL3 (Tee *et al*., 2023), FH9 (Diao *et al*., 2018), and REM1.2 (Huang *et al*., 2019) are expected to identify protein components at the PD-PM. Similarly, MCTPs (Brault *et al*., 2019) and RTNLB6 (Knox *et al*., 2015) are well suited to identify proteins residing at the PD-ER. Additionally, PDCB1 (Simpson *et al*., 2009) and AtBG-ppap (Levy *et al*., 2007) hold the potential for identifying protein complexes localized to the PD-CW. The identification of PD-CW proteomes is particularly compelling, as research on the identity and functions of apoplastic proteins around the PD remains limited. Recently, many cell wall and cell wall-modifying proteins were identified in the high-confidence subset of the *Physcomitrium patens* (HC300) PD proteome (Gombos *et al*., 2023). Successfully mapping the PD-CW proteomes would further validate the utility of PL technologies in identifying apoplastic proteins, offering valuable insights into their roles in plant biology. Overlapping sets of putative interacting proteins could be identified between PD-localized proteins targeting the PD-PM and PD-ER, or the PD-PM and PD-CW, when compared with negative controls (e.g. Col-0 or fluorescent protein–TbID fusions), suggesting the presence of potential core components of the PD proteome. However, a direct comparison between the bait proteins can reveal preferential enrichment of distinct protein subsets. These findings suggest that using a proper control can help fine-map sub-PD proteomes. It is crucial to note that many PD-associated proteins are not exclusively localized to PD and may also be found in other subcellular compartments, such as the PM and ER. Their interactions in these non-PD locations can contribute to variability in the pool of proximally labeled proteins. Nevertheless, PL assays hold significant promises in fine-mapping sub-PD proteomes.

### Identification of PD proteomes in specific tissues/cell types

Using native promoters to express their corresponding proteins will enable the identification of PD protein complexes within the cell types where they are naturally expressed (Fig. 1B). The use of native promoters can yield varying outcomes due to the low activation of the promoters or their cell type-specific activation. To address the challenges posed by cell type specificity, isolating specific cell types to enrich the TbID fusion protein level can be a viable strategy. Alternatively, a promoter with higher activity in the same cell type as the protein of interest can be used. These approaches can enhance the detection of protein interactomes within specific tissues/cell types.

### Mapping dynamic PD proteomes in response to stresses

PD-associated proteins can respond to different stresses. For example, the expression of PDLP5 is up-regulated by bacterial infection (Lee *et al*., 2011) and CRK2 is relocated from the PM to PD during salt stress (Hunter *et al*., 2019). Mapping the dynamic changes in protein interactomes of stress-responsive PD-associated proteins will further reveal the function and regulation of PD during stresses (Fig. 1C).

### Split-TbID to map proteins at PD–PM and PD–ER contact sites

Fusing PL enzymes directly to a bait protein results in labeling at multiple locations where the bait is present. Targeting or anchoring PL enzymes to specific organelles or cellular compartments leads to labeling throughout the entire space within those structures. Split-PL approaches have been developed to enhance spatial and/or temporal specificity, allowing more controlled and precise labeling. Split-TbID is a modified version of TbID where the enzyme is split into two inactive fragments. These fragments can be fused to different proteins or targeted to different cellular compartments, becoming active only when brought together by protein–protein interactions or at contact sites (Cho *et al*., 2020). This technique has been successfully applied to map protein compositions at ER–mitochondria contact sites (Cho *et al*., 2020). A recent study demonstrated that ER–PM apposition at PD is mediated by a protein–lipid tethering complex (Pérez-Sancho *et al*., 2025). Split-TbID could be adapted by targeting the two inactive TbID fragments to PD-PM and PD-ER to identify tether proteins at PD-PM and PD-ER contact sites (Fig. 1D).

### TransitID to map PD-mediated proteome trafficking

Conventional PL methods provide only single time point snapshots of protein interactomes and local proteomes. To capture the short-range proteome trafficking between cells or intracellular trafficking between organelles, Qin *et al*. (2023) developed TransitID (trafficking analysis by sequential incorporation of tags for identification) by multiplexing TbID and APEX2 and performing two PL reactions in tandem within the same biological sample. This method targets TbID and APEX2 enzymes in source and destination compartments or cells, respectively. Small molecule substrates of the TbID and APEX2 (i.e. biotin and alkyne-phenol) are added sequentially into the sample. Dual-labeled proteins can then be identified using MS, providing a detailed map of the transiting proteome. TransitID enables mapping of endogenous protein movement within and between living cells with nanometer spatial resolution (Qin *et al*., 2023). It could be adapted for PD research to map endogenous protein movement between cells through PD (Fig. 1E). APEX2 may not be fully compatible with plant tissues due to high background labeling from endogenous peroxidases and the potential toxicity and stress response induced by the H_2_O_2_ substrate. Other PL systems such as LAccID (Lee *et al*., 2024, Preprint), BLITZ (Xiong *et al*., 2021), and PUP-IT (Liu *et al*., 2018) could replace APEX2 for enhanced adaptability in plant systems.

### Exploring molecular mechanism underlying the trafficking of mobile RNAs

RNA-binding proteins (RBPs) play essential roles in RNA biogenesis, processing, function, and degradation. PL enzymes cannot be directly fused to RNA to map the protein interactome of specific RNAs of interest. To this end, MS2 and Cas13-based PL approaches have been used to identify functionally relevant RBPs for specific mRNAs. An mRNA of interest can be tagged with MS2 RNA stem–loops, and MS2 coat protein (MCP) can be fused with a biotin ligase (Mukherjee *et al.,* 2019; Han *et al*., 2020). When MCP binds to the MS2 loops, the biotin ligase labels RBPs associated with the target mRNA. Adapting this approach for PD research could enable the mapping of RBPs associated with mobile RNAs such as FLOWERING LOCUS T (*FT*; Luo *et al*., 2018) and *KNOTTED1* (*KN1*; Lucas *et al*., 1995) (Fig. 1F). This would shed light on the molecular mechanisms underlying RNA movement and intercellular communication.

By adopting and optimizing these cutting-edge PL technologies, researchers can address key questions about PD structure, function, and dynamics, paving the way for breakthroughs in understanding plant intercellular communication and stress responses.

## Conclusion and future perspective

PL technologies have revolutionized the study of cellular microenvironments, offering powerful tools to map protein networks and dynamics. Applying these technologies to PD biology can bridge existing knowledge gaps, revealing the molecular underpinnings of PD function and regulation. By integrating PL assays with other omics approaches, researchers can elucidate how PD mediate intercellular communication and respond to environmental stimuli, which will enhance our understanding of plant development and stress responses.
